# Identification of Paleo-Events Recorded in the Yellow Sea Sediments by Sorting Coefficient of Grain Size

**DOI:** 10.1371/journal.pone.0044725

**Published:** 2012-09-06

**Authors:** Liguang Sun, Xin Zhou, Yuhong Wang, Wenhan Cheng, Nan Jia

**Affiliations:** 1 Institute of Polar Environment and School of Earth and Space Sciences, University of Science and Technology of China, Hefei, China; 2 Advanced Management Research Center, Ningbo University, Ningbo, China; Plymouth University, United Kingdom

## Abstract

Identification of natural and anthropogenic events in the past is important for studying their patterns and mechanisms; and sensitive proxies in marine sediments are more reliable for identifying these events than those in terrestrial sediments, which are usually disturbed by human activities. Since the main source materials for the sediments in the Northern Yellow Sea Mud are transported by the Yellow River, sedimentary characteristics can be used to reconstruct the historical events that occurred in the Yellow River Valley. In the present study, by analyzing sorting coefficient of grain size in a 250-year sediment core from the Northern Yellow Sea Mud, we identified several major historical events: the Haiyuan Earthquake in AD 1920 and several times of relocation of the Yellow River estuary. The proxy has the potential of detecting and reconstructing historical events; in combination with historical archives, they also provide an accurate dating method.

## Introduction

Identification of natural and anthropogenic events in the past is important for studying their patterns and mechanisms; and sensitive proxies in marine sediments are more reliable for identifying these events than those in terrestrial sediments, which are usually disturbed by human activities.

In China's Near Sea, suspended fine-grained materials are carried by rivers and deposited on the inner shelves to form the mud sediments [1]. Grain size of these sediments is subject to the influence of natural and anthropogenic events in the relevant regions and thus sensitive to various climatic factors. For example, a coarse sand layer found in the muddy sediments is a good indication of storm surge [2]. Grain size of these sediments has been widely used as paleoclimate proxies [2], [3]; however, the historical events recorded in these sediments have attracted much less interests.

The major Yellow River estuary relocation events have been recorded in historical archives [4] and in the sediments transported by the Yellow River. The AD 1855 relocation, one of the largest, has been detected by elements and organic matter δ^13^C changes in the sediments from the southern Yellow Sea [5]. Some other major relocation events have been detected by using sediment facies and total organic carbon / total nitrogen (TOC/TN) changes in a long, but imprecisely dated sediment core from the northern Yellow River [6]. To date, however, no systematic analysis has been performed to identify these events by using sedimentary proxy.

In the present study, we analyzed sedimentary characteristics, including parameters for grain size, magnetic susceptibility (MS) and SiO_2_/Al_2_O_3_ ratio to identify paleo-events recorded in the Northern Yellow Sea Mud (NYSM).

## Materials and Methods

**Figure 1:**
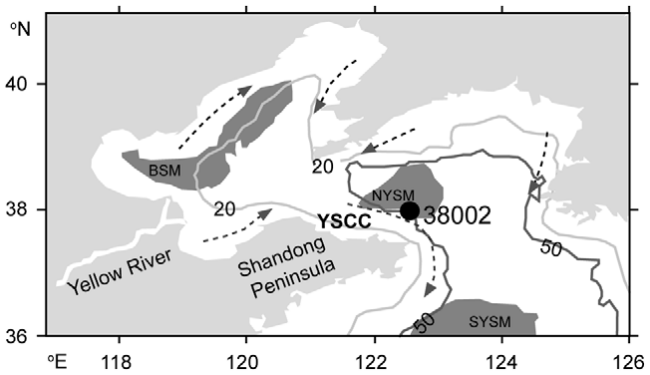
Location of the sampling site (map is modified from [24]). Topographic lines are shown in light grey, mud areas are shown in dark grey, and coastal currents are marked by dashed lines and arrows. BSM: Bohai Sea Mud; NYSM: Northern Yellow Sea Mud; SYSM: Southern Yellow Sea Mud; YSCC: Yellow Sea Coastal Current.

NYSM is located in the north of Shandong Peninsula of China, and its main source materials come from the Yellow River and are transported across the Bohai Bay along the Shandong Peninsula. The Yellow River can transport materials directly to the NYSM region, as shown in the satellite image [7]. In this study, we collected a 34 cm long sediment core, labeled as M38002, from Station 38002 (122°30.21′ E, 37°59.92′ N, water depth 49.2 m; Figure 1) of NYSM by box-corer in 2009 during “The Offshore Sea Opening Research Cruise (Autumn)” on the scientific survey ship “Kexue 1”, Institute of Oceanology, Chinese Academy of Sciences. The permits, from the Institute of Oceanology of Chinese Academy of Sciences were obtained for the described field studies.

**Figure 2:**
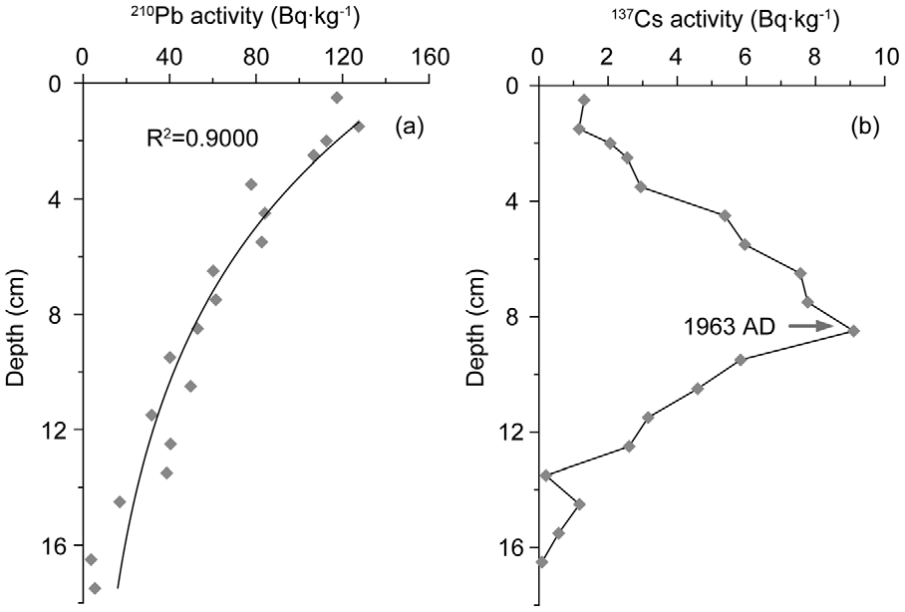
Activity profiles of ^210^Pb (a) and ^137^Cs (b) for the sediment core M38002.

The sediment core was divided at 0.5 cm intervals to collect 68 subsamples, which were analyzed for magnetic susceptibility, grain size, and levels of Si and Al. Chronology of the core is determined by ^210^Pb-^137^Cs dating method (Figure 2).

The samples were pre-treated for grain size analysis. 10–20 ml H_2_O_2_ solution (30%) was added, and the mixture was heated to 100^o^C for 0.5 h to remove organic matter and bathed in 10 ml HCL solution (10%) for 48 h to remove calcareous cement and shell materials. All samples were fully desalted and dispersed by adding 10 ml (NaPO_3_)_6_ (10%) and by ultrasonic treatment of 10 minutes before measurement.

Grain size was measured using Mastersizer 2000 (Malvern Instruments) at the Lab of Soil and Environmental Changes, Taishan University, Taian, China. The measurement range of the instrument is 0.02−2000 μm, the resolution is 0.01 *Φ*, and the repeated measurement error is less than 2%. Measurements of low frequency MS were carried out using the Bartington MS2 susceptibility meter in Nanjing University, Nanjing, China. The absolute contents of SiO_2_ and Al_2_O_3_ were measured using XRF-1800 (X-Ray Fluorescence) Spectrometry (Shimadzu Corporation) in Physical and Chemical Science Experimentation Center of the University of Science and Technology (USTC), Hefei, China.

Radioactivity was measured by germanium detector manufactured by AMETEK Company in the Institute of Polar Environment, USTC, Hefei, China. The samples were dried to constant weight at a temperature of 50 °C, homogenized using a mortar and pestle, and passed through a 120-µm sieve. Samples (between 5–10 g) were then packed into standard counting geometries for gamma analyses and sealed and stored for about one week to allow radioactive equilibration between ^226^Ra and its daughter product ^214^Pb. Spectra were continuously measured for 24 h to obtain enough counts. The resulted spectrum files showed ^210^Pb activity with a peak at 46.5 keV.

## Results and Discussion

### Chronology

The whole core consists of muddy sediments. The upper 23 cm is a light yellow oxide layer, and the lower part dark grey. Some layers contain shell fragments. Because the excess ^210^Pb activity showed a simple exponential relation with depth (Figure 2), typical of the decay curve, indicating a nearly constant sedimentation rate. The ^210^Pb chronology was thus constructed by using a Constant Initial Concentration (CIC) model [8]. The age at 8.5 cm depth, calculated from the ^210^Pb profile, is AD 1961, close to the age of AD 1963 as indicated by the peak value of ^137^Cs profile; thus our ^210^Pb age model appears to be correct. The average sedimentation rate was 0.13 cm·yr^−1^, consistent with the earlier results [9], [10]. The time span of the core is about 254 years (AD 1755–2009), as estimated by extrapolation of the average sedimentation rate.

### Grain size distributions

**Figure 3:**
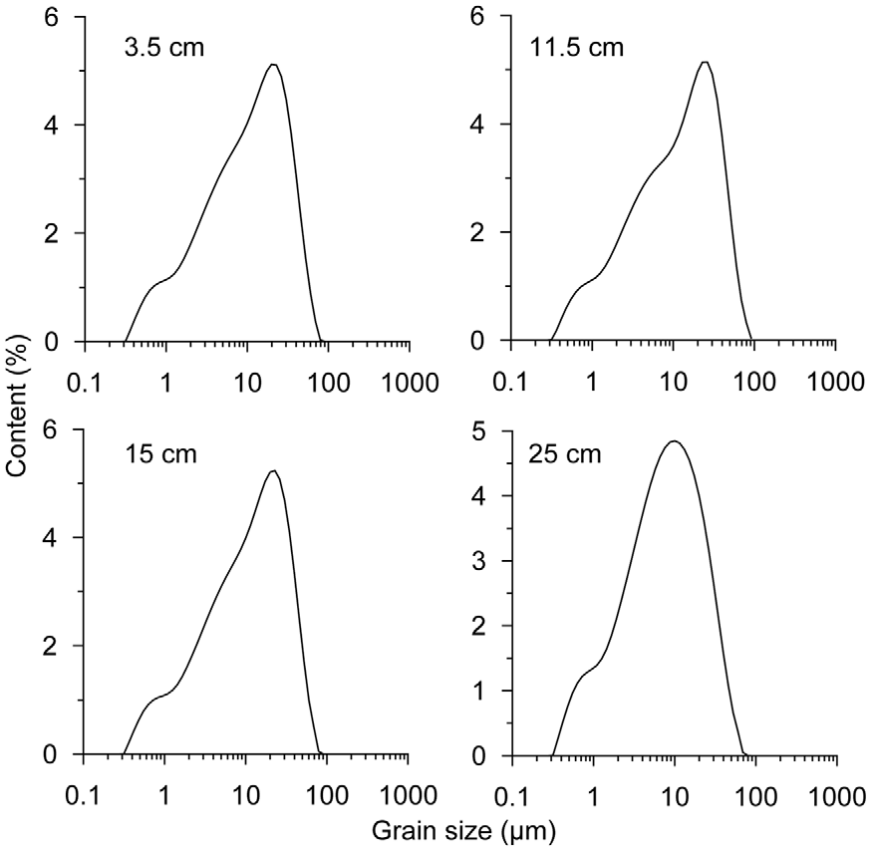
Grain size distributions of four samples selected from the sediment core M38002 at depths of 3.5, 11.5, 15, and 25cm.

Four typical samples were selected from the core M38002 at depths of 3.5, 11.5, 15, and 25cm to study the grain size distributions of the sediments. The frequency distributions (Figure 3) of these four samples are close to log-normal distribution with corresponding skewness of 0.09, 0.08, 0.09, and −0.01, and they are similar to that of the loess [11].

### Identification of events by sorting coefficient for grain size

**Figure 4:**
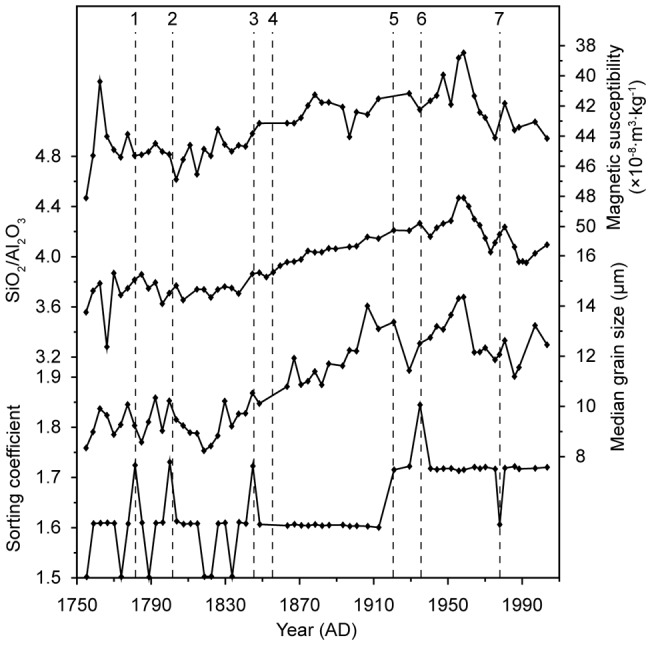
Comparison of sedimentary characteristics of the sediment core M38002 in NYSM with paleo-events. The numbers “1”, “2” and “3” mark the extra-large flood around the Shandong Peninsula in AD 1781, the extra-large flood of Hai and Luan River in AD 1801 and large flood of Liao River in AD 1846, respectively; “4”, “6” and “7” the events of the Yellow river relocation in AD 1855, 1938 and 1976, respectively; “5” the AD 1920 Haiyuan Earthquake.

Median grain size, MS, and SiO_2_/Al_2_O_3_ ratio are usually used as proxies of sedimentary characteristics. The profiles of median grain size and SiO_2_/Al_2_O_3_ ratio in the sediment core M38002 show similar trends during the past 254 years. They have large oscillations without clear trend before AD 1820, gradually rise between AD 1820 and 1960, and then fall abruptly after AD 1960. The MS profile shows an opposite trend (Figure 4).

**Figure 5:**
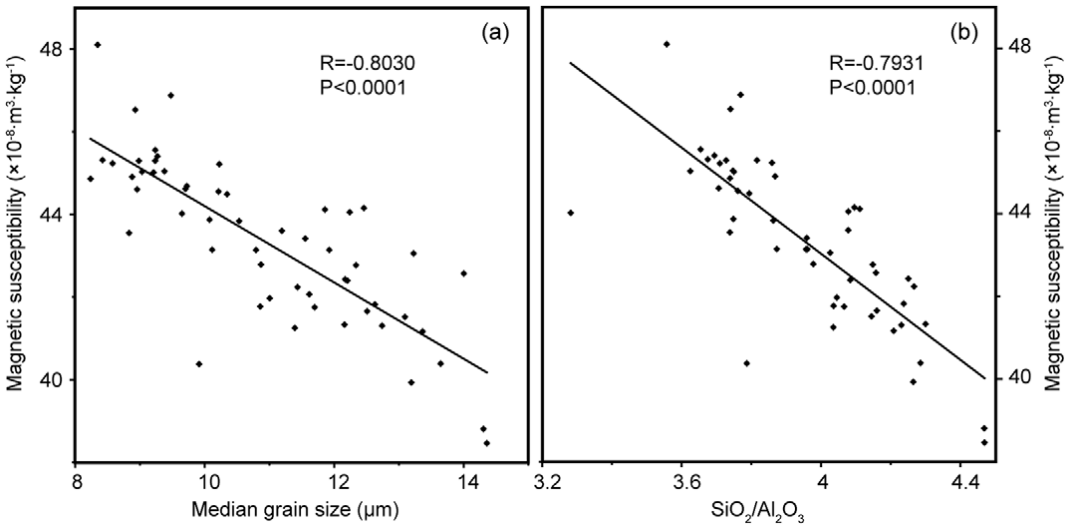
Correlations between MS and the median grain size and the SiO_2_/Al_2_O_3_ ratio of sediment core M38002.

The main source materials of the sediments from the NYSM were originated from the Loess Plateau, and transported by the Yellow River [12]. As expected, the sedimentary characteristics of the core M38002, as shown by the plot of median grain size versus MS and SiO_2_/Al_2_O_3_ ratio (Figure 5), are consistent with those of the loess [13], [14]. Great events, such as relocations of the Yellow River estuary, for the past 254 years have been well documented in historical archives. These events, especially those occurred on the Loess Plateau, could be recorded in the sediments transported by the Yellow River. However, no obvious, corresponding changes in the median grain size, the MS and the SiO_2_/Al_2_O_3_ ratio of the core M38002 could be identified (Figure 4), likely due to relatively homogeneous sedimentary characteristics of the loess.

To search for a proxy that could identify these well-documented events, we examined the link between these events and sorting coefficient (SC). SC, one of the parameters for grain size, has been successfully used as a proxy for hydrodynamic and material source changes, and it can be calculated by two different methods:
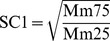
(1)Where Mm_25_ and Mm_75_ are the reaches of the 25th and 75th percentiles using millimeter values [15], respectively, and
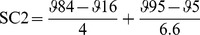
(2)Where φi is the reach of percentile of i by using a φ scale [16].

**Figure 6:**
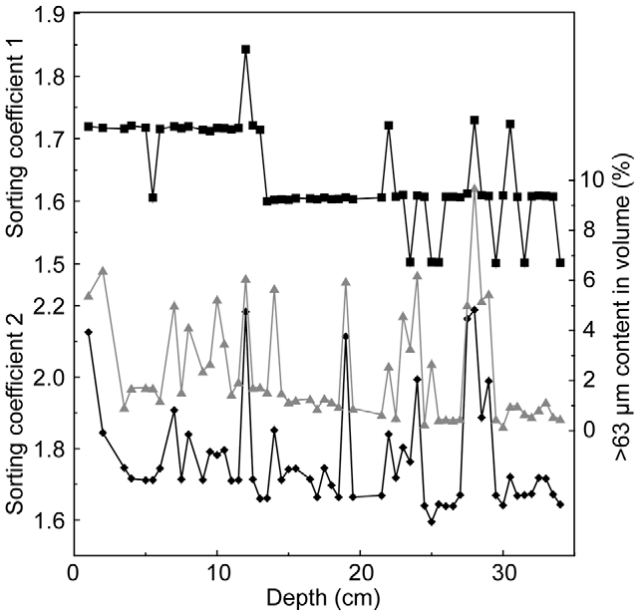
Comparison of sorting coefficients calculated using both Folk & Ward's (SC_2_) [16] and Trask's method (SC_1_) [15]. The concentrations of >63 µm contents are given in volume.

SC_1_ represents changes in the central part of the grain size distribution; therefore it is considered to be insensitive to environmental changes, which are expressed in the tails. SC_2_ is frequently used as a sedimentary characteristic because it covers the tails of a grain size distribution. In our studied region, the source materials are relatively homogeneous, and transportation processes could have significant impacts on the tails of the grain size distribution. The good correlation between SC_2_ and concentrations of >63 µm contents (R = 0.87, P<0.0001; Figure 6) confirmed the impact of transportation processes on SC_2_. Thus, SC_1_, being less sensitive to transportation processes and less noisy, provides a better and more robust proxy of the events occurred in the Yellow River Valley.

The calculated profile of SC_1_ (called SC below) showed three major stages (Figure 4). Before AD 1855, SC varies between 1.50 and 1.73. From AD 1855 to 1920, SC stays stable around 1.60, indicating constant material sources and hydrodynamics. Around AD 1920, SC increases abruptly to around 1.71 and then keeps relatively stable with one peak value of 1.84 around AD 1935 and one bottom value of 1.61 around AD 1977.

**Figure 7:**
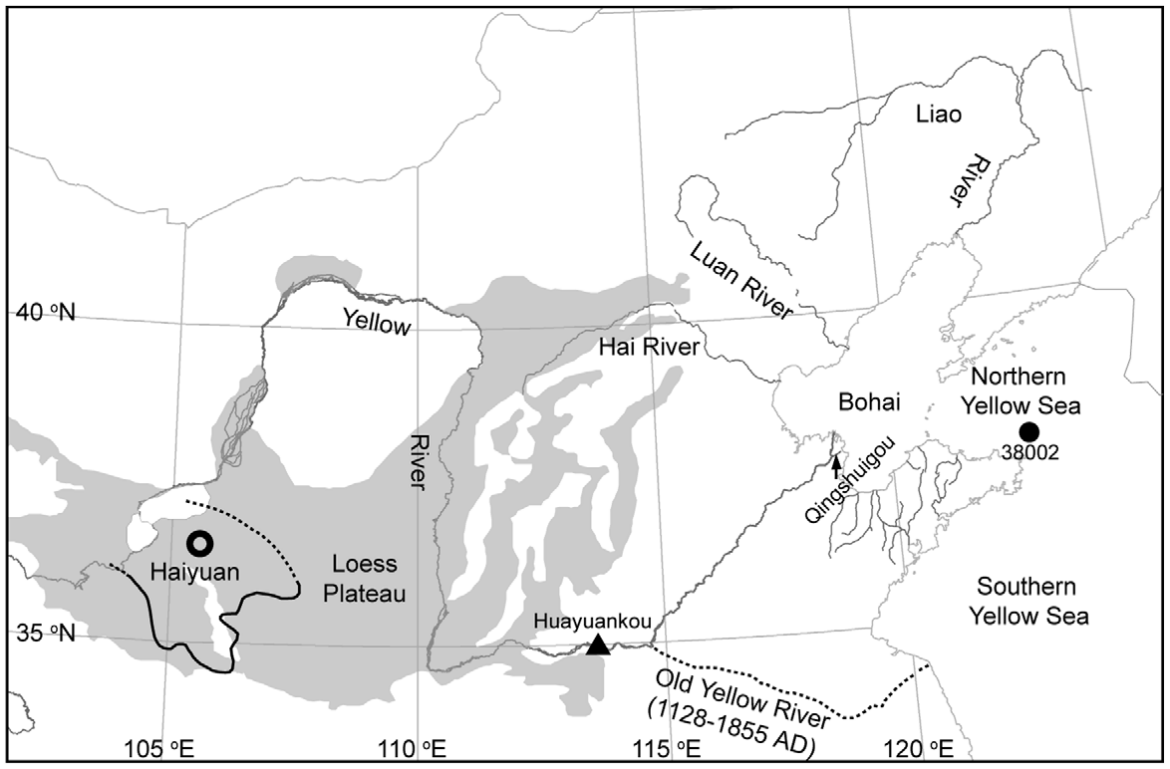
Locations of the Haiyuan County, the Huayuankou, the Old Yellow River, and the Loess Plateau. Areas with seismic intensity of 1920 Haiyuan Earthquake greater than seven degrees on the Chinese Seismic Intensity Scale (modified from [21]) are marked by solid and dashed lines around Haiyuan County; range of the Loess Plateau is modified from [28].

These changes in SC values correspond well to the events documented in historical archives (Figure 4). The Yellow River discharged into the southern Yellow Sea between AD 1128 and 1855 (Figure 7), and then changed to the Bohai Bay in AD 1855 [4]. The Hai River, the Luan River, the Liao River, and the rivers on the Shandong Peninsula provided the main material sources for the sediments in the NYSM before the relocation of the Yellow River estuary from Southern Yellow Sea to the Bohai Bay; thus the large oscillations of SC between AD 1128 and 1855 reflect the hydrodynamic changes of these rivers. Three higher values during these periods coincide with the great flood in the Shandong Peninsula in AD 1781 [17], in the Hai and Luan River regions in AD 1801 and in the Liao River region in AD 1846 [18] (Figure 4).

Large amounts of soil materials from the Loess Plateau are carried by the Yellow River to the sea; the average annual sediment discharge between AD 1952 and 2005 is 7.78 billion tons [19]. After the relocation of Yellow River estuary in AD 1855, the dominant material source of the core M38002 is the Loess Plateau. The abrupt changes of SC around AD 1855 are very likely caused by the relocation of the Yellow River estuary, and the stability of the SC values after that indicates a stable material source supply.

The abrupt increase of SC after AD 1920 concurred with the great Haiyuan Earthquake (Figure 5). This earthquake had a magnitude of 8.5 on the Richter scale, was felt throughout China, caused more than 200,000 deaths, induced many major landslides, changed the topography on the Loess Plateau, and ravined the “Yuan” of loess [20], [21]. Loess is undiagenesised, erosion-prone eolian sediment, so a large amount of loess could be loosed by the great earthquake and transported by the Yellow River into NYSM to cause the increase of SC in the studied sediment core. For the past several decades, human activities may also contribute to the high silt content in the Yellow River [22] and thus the observed SC increase. Additionally, both the East Asian summer and winter monsoons likely had abrupt changes around AD 1920; but they are unlikely the cause for abrupt increase of SC after AD 1920 since they have opposite impacts on the hydrodynamics at the 38002 Station and their strength have been oscillating even since [23], [24].

The peak of SC around AD 1940 coincided with the blast of the Yellow River Dyke in AD 1938 and the great flood in AD 1939. During the China's Anti-Japanese War, the Chinese army blew up the dyke at Huayuankou (Figure 7) in AD 1938, causing the relocation of the Yellow River estuary to the southern Yellow Sea during AD 1938 to 1946 [4]. The relocation of the Yellow River estuary changed the main source materials of M38002 from the Yellow River to other rivers. The great flood (the largest flood since AD 1801) at Hai River Basin (Figure 4) in AD 1939 [25] dramatically increased the soil material supply to these rivers and very likely contributed to the SC increase.

The sharp drop of SC around AD 1978 is very likely linked to the relocation of the Yellow River outflow to Qingshuigou River in AD 1976. The event resulted in a large and rapid accumulation of muddy/sand material in the subaqueous delta near the estuary until AD 1980 [26]. After AD 1980, the accumulation rate decreased abruptly and then remained stable up to now [27], so the SC bounded back and stayed stable.

## Conclusions

In the present study, by analyzing grain size, MS and SiO_2_/Al_2_O_3_ ratio in a 254-year sediment core from NYSM, we identified several historical events: the Haiyuan Earthquake in AD 1920 and several times of relocation of the Yellow River estuary. Different proxies have different sensitivities to different geological and environmental events. SC seems to be less noisy and more robust, and as a new method, it has a good potential of detecting earlier events. Furthermore, in combination with accurate historical archives, the SC proxy from the sediments of relatively homogenous material sources can provide additional dating control for sediment cores.
